# Unveiling the neuroinflammatory pathogenesis of persistent functional dyspepsia in *H. pylori* infection: Insights on MMP‐9 as a therapeutic target

**DOI:** 10.1002/ctm2.1456

**Published:** 2023-10-29

**Authors:** Yujen Tseng, Lingxi Lin, Shaocong Mo, Suhan Zhao, Qiwei Shen, Huan Song, Haoshu Cui, Jun Zhang, Wanwei Zheng, Zhongguang Luo, Feifei Luo, Jie Liu

**Affiliations:** ^1^ Department of Digestive Diseases Huashan Hospital Fudan University Shanghai China; ^2^ Department of General Surgery Huashan Hospital Fudan University Shanghai China

Dear Editor,

Persistent functional dyspepsia even after successful eradication of *Helicobacter pylori* infection severely impairs the quality of life for affected individuals.^1^ Although helicobacter pylori (*H. pylori*) is a non‐invasive bacterium, it stimulates a robust inflammatory response that evokes persistent and progressive gastric mucosal inflammation and gastric neurocirculatory disturbances.

The enteric nervous system (ENS) possesses the ability to control gastrointestinal function independent of the central nervous system (CNS).^2^ Subsequent myenteric neuronal damage and apoptosis may further contribute to persistent dyspeptic symptoms due to diminished motor and secretory functions of the stomach, even after *H. pylori* eradication.

Currently, few studies have investigated the direct neurotoxic effect of *H. pylori* on a local spectrum. The present study is aimed at further investigating the role of *H. pylori* infection on neuronal homeostasis from a regional perspective, especially that of the gastric enteric nervous system.

From January 2020 to December 2022, a total of 434 patients treated for *H. pylori* infection received assessment for dyspepsia symptoms and quality of life via the Nepean Dyspepsia Index‐Short Form (NDI‐SF).^3^ The average score (range 2−10) for each sub‐scale were 3.96, 4.01, 5.06, 4.37 and 3.89 for the tension, interference, eating/drinking, knowledge/control and work/study, respectively. The average NDI‐SF score was 21.30. Interestingly, we noted 38% of patients had an NDI‐SF score of ≥25 points. This preliminary clinical data suggest that a substantial portion of patients have persistent dyspeptic symptoms even after *H. pylori* eradication (Table 1).

**TABLE 1:** Characteristics of patients who received *H. pylori* eradication and assessment of functional dyspepsia symptoms.

	Global population	NDI‐SF ≥ 25	NDI‐SF < 25	
	*N* = 434	*N* = 132	*N* = 302	p‐Value
**Clinical characteristics**				
Gender				.859
Male	205 (47.2%)	61 (46.2%)	144 (47.7%)	
Female	229 (52.8%)	71 (53.8%)	158 (52.3%)	
Age	44.4 (13.3)	45.2 (13.0)	44.1 (13.4)	.408
Gastritis				.028
Mild	163 (37.6%)	54 (40.9%)	109 (36.1%)	
Moderate	262 (60.4%)	72 (54.5%)	190 (62.9%)	
Severe	9 (2.07%)	6 (4.55%)	3 (.99%)	
Inflammatory activity				.672
Absence	156 (35.9%)	45 (34.1%)	111 (36.8%)	
Presence	278 (64.1%)	87 (65.9%)	191 (63.2%)	
Atrophy				.268
Absence	360 (82.9%)	105 (79.5%)	255 (84.4%)	
Presence	74 (17.1%)	27 (20.5%)	47 (15.6%)	
Metaplasia				.279
Absence	338 (77.9%)	98 (74.2%)	240 (79.5%)	
Presence	96 (22.1%)	34 (25.8%)	62 (20.5%)	
**NDI‐SF subscale**				
Tension	3.96 (1.54)	4.52 (1.43)	3.72 (1.52)	<.001
Interference	4.01 (1.74)	4.95 (1.84	3.59 (1.52)	<.001
Eating/Drinking	5.06 (2.47)	7.62 (1.92)	3.93 (1.74)	<.001
Knowledge	4.37 (1.92)	5.94 (1.70)	3.69 (1.58)	<.001
Work/Study	3.89 (1.41)	4.54 (1.28)	3.61 (1.37)	<.001
**NDI‐SF total**	21.3 (5.26)	27.6 (2.64)	18.5 (3.45)	<.001

Abbreviation; NDI‐SF, Nepean Dyspepsia Index‐Short Form.

Gastric mucosal tissue samples were obtained from patients with *H. pylori* infection and healthy control during gastroscopy examination. The iDISCO method with tissue clearing immunofluorescence showed a 3D reconstruction of disrupted neural network structure in *H. pylori* infection with a decrease in neuron density, compared to normal control.^4^ Immunofluorescent staining with PGP9.5 also showed a decrease in neuronal fiber density, especially in the muscularis mucosae and lamina propria in *H. pylori* infected patients (Figure 1). The xCell method showed the abundance of neurons was significantly decreased in *H. pylori* infected patients.

**FIGURE 1:**
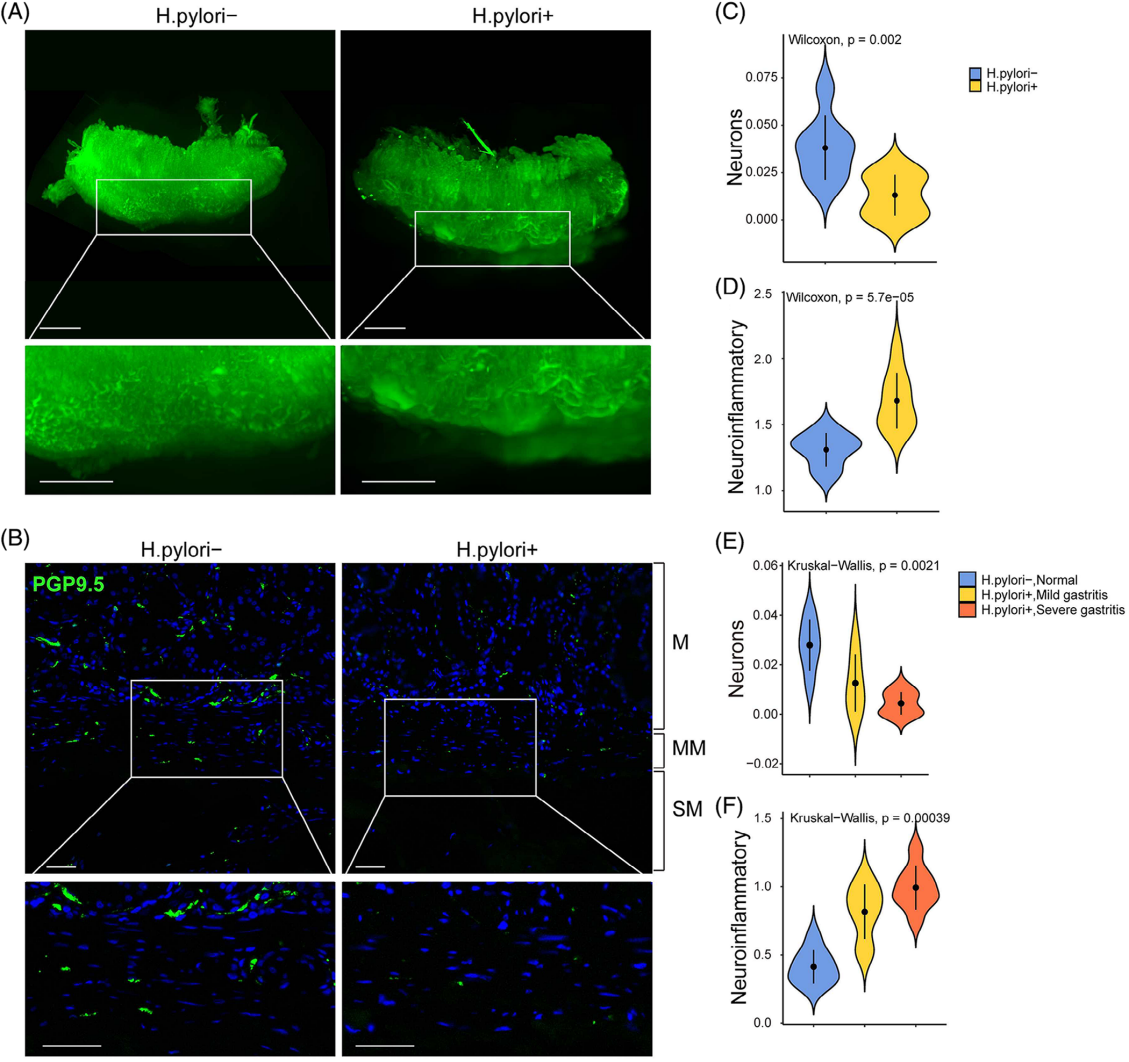
Disruption of gastric ENS in H. pylori infection. (A) Tissue clearing of gastric mucosal biopsy showed a disruption in the neural structure in H. pylori infection compared to normal control; (B) Full‐thickness immunofluorescent staining showed a decrease in PGP9.5 staining in H. pylori infection in the muscularis mucosae layer; (C) A significant decrease in neuron density and neuroinflammatory score was noted in H. pylori infection; (D) external validation confirmed a significant decrease in neuron density and neuroinflammatory score in different degrees of H. pylori gastritis, compared to normal control. H. pylori, Helicobacter pylori.

A significant enrichment in neuroinflammatory response gene set (GO: 0150076) was noted in *H. pylori* infected patients, with a significant increase in neuroinflammatory score. Validation with an external dataset (GSE60427) confirmed that neuron density decreased with severity of gastritis, while neuroinflammatory score was concurrently increased.

Among the 31 neuroinflammatory genes, a total of 12 genes were significantly upregulated in the *H. pylori* infected group. In which, Matrix metalloproteinase‐9 (MMP‐9), Signal Transducing Adaptor Family Member 1, Tumor Necrosis Factor and Cathepsin C showed the highest correlation with *H. pylori* infection. Based on previous results, MMP‐9 was identified as the most relevant neuroinflammatory response gene associated with *H. pylori* infection (Figure 2A). A single‐cell RNA sequencing dataset GSE134520 demonstrated that MMP‐9 was mainly expressed in the macrophage cluster (Figure 2B).^5^ Subsequent immunofluorescent staining of gastric mucosal biopsy samples showed colocalization of MMP‐9 and macrophages stained with CD11b (Figure 2C).

**FIGURE 2:**
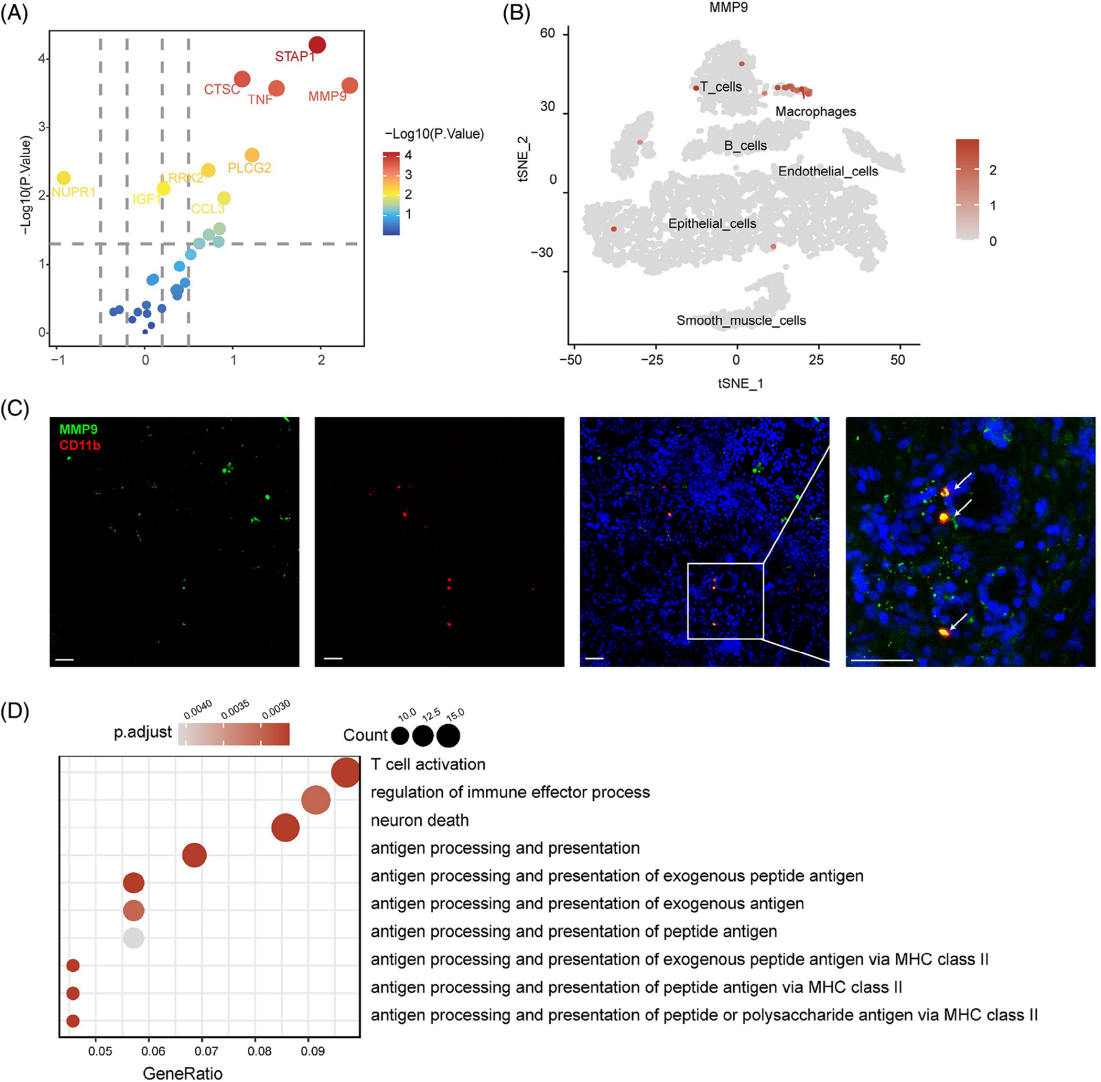
MMP‐9 was mainly expressed in macrophages. (A) Significant differentially expressed genes (DEGs) in H. pylori infection; (B) Single cell sequencing identified the cluster of MMP‐9 in macrophages; (C) Immunofluorescence staining of MMP‐9 and CD11b, scale bar 30 µm; (D) Association between MMP‐9 and macrophages; (E) KEGG pathway analysis of DEGs in MMP+ macrophages and MMP9‐macrophages. H. pylori, Helicobacter pylori; MMP‐9, Matrix metalloproteinase‐9.

Gene Ontology (GO) enrichment analyses demonstrated that macrophage with MMP‐9+ expression was highly correlated with T‐cell activation, regulation of immune effector process and neuron death (Figure 2D), which established the critical role of MMP‐9 in neuron injury in the evolution of *H. pylori* induced gastritis (Figure 2).

To confirm the expression of MMP‐9 can be instigated by *H. pylori* infection, an ex vivo cell culture of THP‐1 was treated with different multiplicity of infection (MOI), which confirmed a significant upregulation of MMP‐9 expression after *H. pylori* infection (Figure 3A). Gelatin zymography of THP‐1 supernatant also confirmed the enhancement of MMP9 secretion after *H. pylori* infection, which was suppressed after addition of MMP9 inhibitor SB‐3CT (Figure 3B). To further examine the molecular identity of *H. pylori* induced neuroinflammation, the supernatant of preconditioned THP‐1 was added to SH‐SY5Y cell culture to mimic the immune response after *H. pylori* infection. Flow cytometry showed an increase in Annexin V+/7‐AAD‐ early apoptotic cells and Annexin V+ total apoptotic cells, with an overall decrease in cell viability. Cell apoptosis was significantly suppressed after addition of SB‐3CT, indicating that *H. pylori* induced neuron apoptosis was macrophage‐derived MMP‐9 dependent (Figure 3C‐D). Full‐thickness immunofluorescent staining of gastric sample showed proximal localization of macrophages and nerve fibers in lamina propria (Figure 3E), which suggested that the neuroinflammatory response in *H. pylori* infection is induced by macrophages.

**FIGURE 3:**
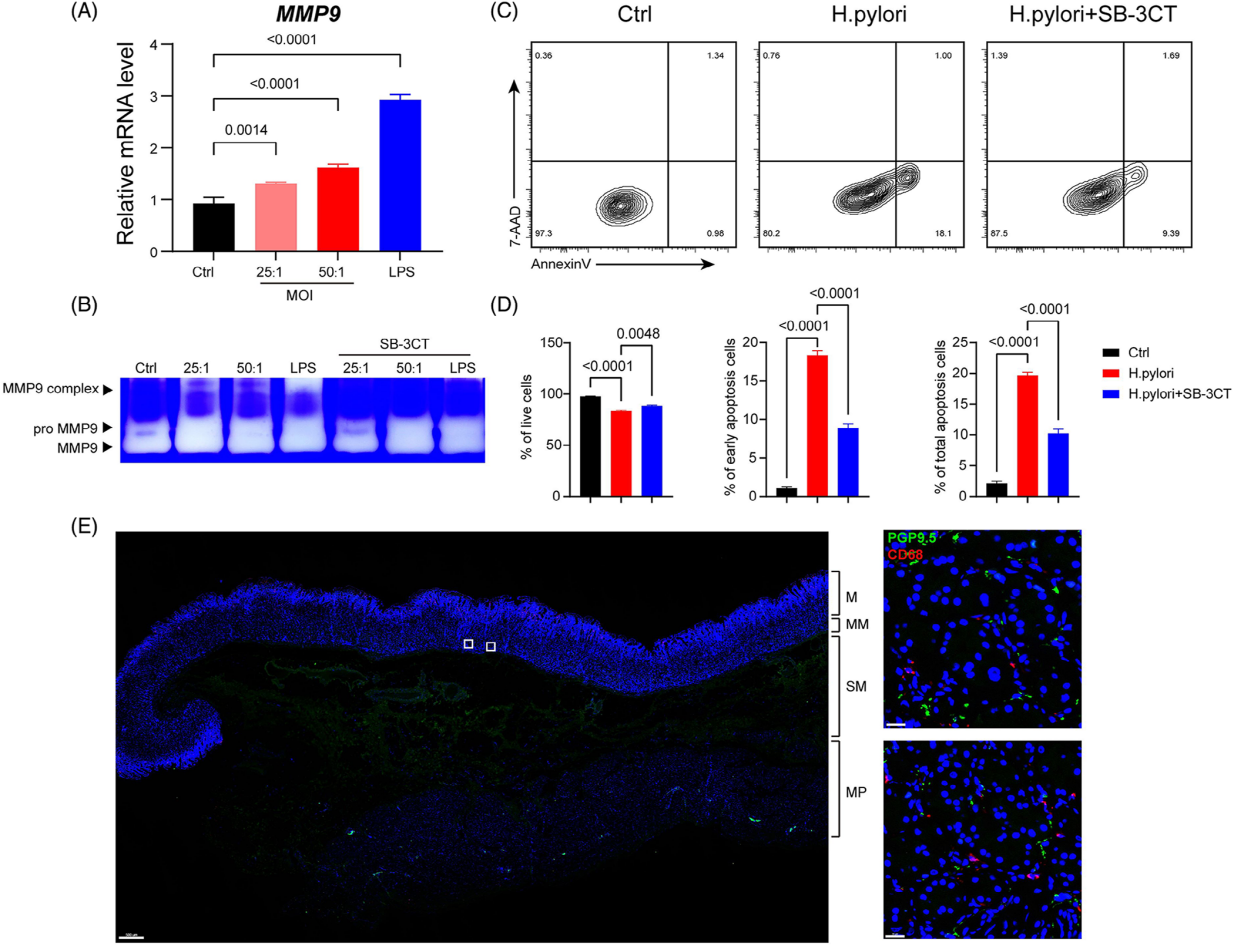
H. pylori infection induced apoptosis of SH‐SY5Y through upregulation of MMP9 in macrophages. (A) Expression of MMP‐9 in THP‐1 cells after H. pylori infection; (B) Zymography of supernatant from infected THP‐1 cell, SB‐3CT (20 μM) were added before infection; (C) (D) SH‐SY5Y cells were treated with supernatant from infected THP‐1 cells, cell viability of SH‐SY5Y cells was measured by AnnexinV‐7AAD staining; (E) Full‐thickness Immunofluorescence staining of PGP9.5 and CD68, M:Mucosa, MM: Muscularis Mucosa,SM: Submucosa, MP: Muscularis Propria, scale bar (left) 500 µm, scale bar (right) 20 µm. H. pylori, Helicobacter pylori; MMP‐9, Matrix metalloproteinase‐9.

The role of *H. pylori* infection in functional dyspepsia is well established. However, insufficient resolution of dyspeptic symptoms after *H. pylori* eradication is undoubtedly observed in clinical practice. Our data demonstrated persistent of severe dyspeptic symptoms (NDI‐SF ≥ 25) in approximately 30% of patients who received *H. pylori* eradication, which prompted further investigation of the underlying mechanism.

The pathogenesis of function dyspepsia is multifactorial, and the involvement of the enteric nervous system has recently been explored. Since, symptoms of dyspepsia are often a consequence of motility disorder, sensorimotor dysfunction presenting as hypersensitivity to mechanical or chemical stimuli, immune activation, can be summated to disorders of the autonomic and enteric nervous system.^6^ In the present study, different techniques such as, tissue clearing, wholemount staining, immunohistochemistry, RNA‐sequencing and transpose convolution machine learning were utilized to confirm disruption in the gastric neuronal network and decrease in neuron density after *H. pylori* infection. The neuroimmune crosstalk may potentially explain the persistent symptoms of dyspepsia in patients after *H. pylori* eradication.

*H. pylori* infection is a multifaceted inflammatory process that involves different aspects of pathogen virulence factors, host immune responses, and colonizing capacity in new host niches. Even with successful eradication, symptoms of local inflammation may sometimes persist. In essence, our findings demonstrated evidence of neuron damage in *H. pylori* infection. Among which MMP‐9 was most significantly correlated with neuroinflammation and was found to be highly expressed in macrophages. MMP‐9 has displayed immunoreactivity which instigated macrophages‐mediated neuron apoptosis. Previously, MMP‐9 has also been reported to participate in the pathology development of gastric cancer.^7^ These findings provide a novel point of target to prevent excessive neuroinflammation during *H. pylori* infection, which may be the underlying cause for persistent dyspeptic symptoms even after *H. pylori* eradication. Emphasis on neuroprotection may shed light to a different treatment approach in functional dyspepsia and *H. pylori*c infection.

## FUNDING INFORMATION

This work was supported by the National Natural Science Foundation of China (grant numbers: 82121002 and 82173186), Shanghai Rising‐Star Program (grant number: 22QA1401600).

## Supporting information


Supporting Information

## References

[1] Ford AC , Tsipotis E , Yuan Y , Leontiadis GI , Moayyedi P . Efficacy of Helicobacter pylori eradication therapy for functional dyspepsia: updated systematic review and meta‐analysis. Gut. 2022;71(10):1967‐1975 10.1136/gutjnl-2021-326583 35022266

[2] Spencer NJ , Hu H . Enteric nervous system: sensory transduction, neural circuits and gastrointestinal motility. Nat Rev Gastroenterol Hepatol. 2020;17(6):338‐351. 32152479 10.1038/s41575-020-0271-2 PMC7474470

[3] Talley NJ , Verlinden M , Jones M . Quality of life in functional dyspepsia: responsiveness of the Nepean Dyspepsia Index and development of a new 10‐item short form. Aliment Pharmacol Ther. 2001;15(2):207‐216. 11148439 10.1046/j.1365-2036.2001.00900.x

[4] Renier N . et al., iDISCO: a simple, rapid method to immunolabel large tissue samples for volume imaging. Cell. 2014;159(4):896‐910. 25417164 10.1016/j.cell.2014.10.010

[5] Zhang P , Yang M , Zhang Y , Xiao S , Lai X , Tan A , Du S , Li S . Dissecting the Single‐Cell Transcriptome Network Underlying Gastric Premalignant Lesions and Early Gastric Cancer. Cell Reports. 2019;27(6):1934‐1947.e5. 10.1016/j.celrep.2019.04.052 31067475

[6] Sticlaru L . et al., Neuroimmune cross‐talk in Helicobacter pylori infection: analysis of substance P and vasoactive intestinal peptide expression in gastric enteric nervous system. J Immunoassay Immunochem. 2018;39(6):660‐671. 30325259 10.1080/15321819.2018.1529683

[7] Sokolova O , Naumann M . Matrix Metalloproteinases in Helicobacter pylori‐Associated Gastritis and Gastric Cancer. Int J Mol Sci. 2022;23(3). 10.3390/ijms23031883 PMC8836485 35163805

