# Supplementary material for: Unveiling the neuroinflammatory pathogenesis of persistent functional dyspepsia in H. pylori infection: Insights on MMP‐9 as a therapeutic target
Source: Clin Transl Med. 2023 Oct 29;13(11):e1456. doi: 10.1002/ctm2.1456 (PMC10613753; doi:10.1002/ctm2.1456)
Supplement: Supplementary file 1 — Supporting Information [file CTM2-13-e1456-s001.docx]

**Methods**

Patient Population

Patients diagnosed and treated for *H. pylori* infection at a tertiary center in Shanghai, China, were prospectively enrolled. Enrollment criteria included: 1. *H. pylori* infection confirmed by C^13^-Urea breath test (CUBT-13) or rapid urease test (RUT), 2. Patients who received *H. pylori* eradication treatment 3. Patients fulfilled the diagnosis of function dyspepsia based on the Rome IV criteria. 4. Received upper GI endoscopy to rule out evidence of structural disease. All patients were given Nepean Dyspepsia Index-Short form (NDI-SF) at least 2 weeks after *H. pylori* eradication.

Assessment of Functional Dyspepsia via Nepean Dyspepsia Index – Short form (NDI-SF)

The Nepean dyspepsia index – short from (NDI-SF) was derived from the original Nepean Dyspepsia Index (NDI) with 42 items, which contains 5 sub-scales examining different domains including tension/anxiety, interference with daily activities, disruption to regular eating/drinking, knowledge towards/control over disease symptoms and interference with work/study. Each sub-scale contains two questions and were measure by a 5-point Likert scale ranging from 0 (not at all or not applicable), 1 (a little), 2 (moderately), 3 (quite a lot), to 4 (extremely). Patients who have received successful *H. pylori* eradication were given the NDI-SF assessment at our outpatient clinic by a gastrointestinal specialist.

Sample collection and data acquisition

A total of 23 gastric biopsy samples were obtained from patients who received gastroscopy examination at Huashan Hospital, Fudan University. Tissue samples were further divided into *H. pylori* infection or normal control based on CUBT-13 or RUT results (16 *H. pylori* infected patients vs. 7 normal control). Patients provided informed consent for access to their clinical data and donation of mucosal biopsy sample used for rapid urease test (normally discarded as medical waste). GSE60427, containing microarray expression profiling of gastric epithelium with *H. pylori* infection, was downloaded from GEO database (https://www.ncbi.nlm.nih.gov/geo/) to verify the results. GSE134520, containing single-cell RNA sequencing (scRNA-seq) of the gastric mucosa of patients with *H. pylori* related gastritis was also obtained from GEO database. scRNA-seq data underwent standard Seurat pipeline, including data filtering, dimensionality reduction and clustering. Cell types were annotated with known cell markers of gastric mucosa.

RNA sequencing

Total RNA was extracted from the samples with Trizol reagent (Invitrogen, California, USA). RNA integrity was detected by agarose gel electrophoresis. RNA purity was detected by Nanodrop (Thermo Fisher Scientific, Massachusetts, USA) and RNA concentration was quantified by Qubit (Thermo Fisher Scientific, Massachusetts, USA). mRNA was enriched with magnetic beads with Oligo (dT) and was randomly broken into short fragments of about 200 bp. cDNA library was constructed using VAHTS Stranded mRNA-Seq Library Prep Kit (NR602, Vazyme, Jiangsu, China). Sequencing was performed using the Illumina Nova6000 system.

The raw data quality was evaluated using fastQC. The raw data was processed and filtered using trim_galore and was aligned with Hisat2, quantified by featureCounts. Gene expression was finally determined by Transcript per Kilobase per Million mapped reads (TPM).

Machine learning

To illustrate the ability of neuroinflammatory genes to discriminate between *H. pylori* positive and negative mucosa, we first screened variables by random forest. Genes with a Gini coefficient greater than 0 were included.

Gene set enrichment and immune cell infiltration

Neurons in the samples were calculated using *xCell* R package, with TPM expression matrix as the input data. Furthermore, Neuroinflammation gene set was obtained from GSEA-Msigdb (https://www.gsea-msigdb.org/gsea/, GO:0150076) and neuroinflammation score was assessed using *GSVA* package. The proportion of immune cell infiltration was deconvoluted using CIBERSORT method. GSEA, GO enrichment analysis were done by clusterProfiler.

iDISCO Tissue Cleaning

Six gastric biopsy samples were obtained from patients who underwent gastroscopy (3 *H. pylori* infected patients vs. 3 normal control). Tissue cleaning was conducted as previously described. After fixation with 4% paraformaldehyde, clinical samples were dehydrated through a series of 20%, 40%, 60%, 80%, and 100% methanol solutions. Samples were then further incubated overnight in a 66% dichloromethane/33% methanol solution, followed by two washes with methanol before being bleached in an ice-cold 5% H2O2 methanol solution. To rehydrate, samples were exposed to a series of gradient methanol solutions starting from 20% and reaching 100%. For permeabilization, a 0.2% Triton X-100/20% DMSO/0.3 M glycine solution was prepared, and samples were blocked in a solution of 6% donkey serum, 10% DMSO, and 0.2% Triton X-100 for 2 days. After washing for 2 hours, samples were incubated in a primary antibody solution containing anti-NF-M/H (1:300) for four days. Following this, samples were washed and cultured in a secondary antibody solution for another four days.

For tissue cleaning, samples were first dehydrated in gradient methanol solutions, then incubated in a 66% dichloromethane/33% methanol solution for 3 hours, followed by 100% dichloromethane for 30 minutes, and finally in 100% dibenzylether overnight.

Cell culture

THP-1 and SH-SY5Y cell lines were purchased from ATCC. THP-1 cells were grown in Roswell Park Memorial Institute (RPMI) 1640 medium containing 10% fetal bovine serum (Thermo Fisher Scientific USA), 100 U/ml penicillin, and 100 mg/ml streptomycin. PMA treatment was applied to THP-1 cells for subsequent experiments. SH-SY5Y cells were grown in Dulbecco's Modified Eagle's Medium (DMEM) containing 10% fetal bovine serum (Thermo Fisher Scientific, USA), 100 U/ml penicillin, and 100 mg/ml streptomycin. All cell lines were cultured at 37 °C under 5% CO2.

*H. pylori* culture and infection

The *H. pylori* strain (26695) used in this study was gifted from Renji Hospital, Shanghai Jiaotong University. The bacteria were cultured on Columbia agar plates at 37°C under microaerophilic conditions. After 48 hours, they were collected and resuspended in phosphate-buffered saline (PBS) at a concentration that was determined to yield a multiplicity of infection (MOI) of 25 when added to THP-1 cells. Prior to infection, the THP-1 cell medium was changed to serum-free and antibiotic-free medium. Twelve hours post-infection, cell supernatants were assayed for producing MMP-9 by gelatin zymography.

Gelatin zymography

THP-1 cell supernatants were mixed with non-reducing sample dilution buffer (2.5% SDS) and treated on ice for 1 hour before analytical separation in 0.1% gelatin gels by zymography. After electrophoresis, gels were washed twice for 30 minutes using washing buffer (2.5% Triton X-100, 50mM Tris-HCl, 5mM CaCl2, 1μM ZnCl2, pH=7.5) to remove SDS and incubated for 16 hours in collagenase buffer (1% Triton X-100, 50mM Tris-HCl, 5mM CaCl2, 1μM ZnCl2, pH=7.5). The gels were then stained with a solution of 40% methanol, 10% acetic acid, 0.5% Coomassie Blue, and destained until clear bands could be identified. Three independent experiments were performed.

Real time PCR

THP-1 cells were infected with *H. pylori* for twelve hours before RNA extraction. Total RNA of THP-1 cells were extracted using RNAiso PLUS (Takara, Japan), PrimeScript™ RT reagent Kit (Takara, Japan) was used to synthesis complementary DNA. The expression of MMP-9 was measured by quantitative PCR with the following primers:

MMP9 Forward Primer (5′−3′) : TGTACCGCTATGGTTACACTCG

MMP9 Reverse Primer (5′−3′) : GGCAGGGACAGTTGCTTCT

Apoptosis detection by flow cytometry

SH-SY5Y cells were harvested 24 hours after treating by filtered supernatant from infected THP-1 cells. Cells were resuspended in binding buffer containing Annexin-V (Thermo Fisher Scientific, USA) and 7-AAD (Thermo Fisher Scientific, USA) for 15 min. All samples were quantified by flow cytometry (Beckman Coulter, USA) within an hour. All experiments were performed in triplicates.
